# The Relationship between Eating and Lifestyle Habits and Cancer in Van Lake Region: Another Endemic Region for Esophageal and Gastric Cancers

**DOI:** 10.1155/2015/254823

**Published:** 2015-01-15

**Authors:** Sebahattin Celik, E. Murat Yılmaz, Ferhat Özden, Cetin Kotan, Hayrettin Okut

**Affiliations:** ^1^General Surgery Department, Faculty of Medicine, Van Yüzüncü Yıl University, 65080 Van, Turkey; ^2^General Surgery Clinic, Van Regional Training and Research Hospital, 65100 Van, Turkey; ^3^Pathology Clinic, Van Regional Training and Research Hospital, 65100 Van, Turkey; ^4^Department of Biometry and Genetics, Faculty of Agriculture, Van Yüzüncü Yıl University, 65080 Van, Turkey

## Abstract

*Purpose*. To examine the relationship between esophageal and gastric cancers commonly seen in Van Lake region and the traditional eating habits of the geography.* Materials and Methods*. Esophageal and gastric cancer cases, who underwent surgery between January 1, 2012, and December 31, 2013, were examined. Pathology reports of the patients and presence of* Helicobacter pylori (HP) *were recorded. Surveys were filled by face to face meeting or telephone call. Control group was created with randomly selected individuals without any cancer diagnosis having age, gender, and socioeconomic characteristics similar to patient group. All data were analyzed using SAS.9.3 statistical programme.* Results*. Compared with the control group, herby cheese consumption (a component of eating habits) and smoking were significantly higher in the patient group (*P* < 0.001). Tandoor exposure is compared in terms of female gender, and significant difference was found between the groups (*P* = 0.0013). As a result of the analysis with logistic regression more than 150 gr of herby cheese consumption per day was found to increase the cancer risk (odds ratio 1.017; 95% CI: 1.012–1.022).* Conclusion*. A high consumption of herby cheese, cooking bread on tandoor, and heavy smoking were seen to be important risk factors for esophageal and gastric cancers.

## 1. Introduction

Regional differences and inequalities in diseases are much more clear in cancer cases which can be seen as reflections of worldwide socioeconomic, politic, and environmental inequalities. According to GLOBOCAN 2012 data, 8.2 million people die from cancer worldwide and more than half of them (64.9%) are in less developed countries [1]. 70% of deaths from gastric and esophageal cancers, which rank 3rd and 6th in the cancer deaths worldwide, respectively, are seen in developing countries including our country [1, 2]. According to 2011 data from the Ministry of Health, esophageal cancer is not one of the 10 most common cancers in Turkey, whereas the incidence of gastric cancer is 8.6 in females and 21.6 in males per 100.000 per year [3]. In the eastern region of Turkey, especially in Van Lake region, yet there are no serious epidemiological studies; most recently in 2006, in a study conducted in 1584 cancer patients, Alıcı et al. reported that gastric cancer (26.5%) and esophageal cancer (15.8%) rank first and second most common cancers, respectively [4]. Regional differences in gastric and esophageal cancers both in the same country and around the world often have been linked to eating and lifestyle habits and frequency of* HP* infection [1, 5, 6]. Studies made in geographies, known as* “the esophageal cancer belt or line” (which if somewhat extended, Eastern Anatolian Region can be included)* that connect the high-risk zones for esophageal cancer* (China's central northern region, Central Asian Republics, Iran's northern parts)*, have put emphasis on, in particular, eating and drinking very hot foods and drinks, consumption of smoked and brined products, lack of fresh fruit and vegetable consumption, and smoking and those studies reported significant risks [2, 7, 8].

Lifestyles and eating habits of the people in Van Lake region,* one of the endemic areas for gastric and esophageal cancers*, are peculiar to this geography; besides they are similar in risky regions mentioned before. In this study we aimed to evaluate the relationship between esophageal and gastric cancers and* HP* infection, herby cheese consumption, drinking hot tea, cooking bread on tandoor, and smoking which were seen as potential risks and recommended to be investigated in etiopathogenesis in the previous studies conducted in here, Van Lake region [4, 9].

## 2. Materials and Methods

Van Regional Training and Research Hospital (VTRH) and Yüzüncü Yıl University Faculty of Medicine Hospital (YYU-FMH) are the most important medical centers serving Van and all the provinces of Van Lake region. After getting VTRH Ethics Board approval, both hospitals' records were used. The study was planned as a case-control study and it includes the pathologic reports of the patients who underwent surgery in VTRH and YYU-FMH for two years between January 1, 2012, and December 31, 2013. The control group comprised randomly selected individuals, who were admitted to hospital for reasons other than cancer, with socioeconomic profile, age, and sex similar to those of the patient group. Therefore when setting the control group, we have made a selection from the nearest population of patients in the study group. Our inclusion criteria for control group were being in a similar socioeconomic status with study group, being in a same age group, and living in close proximity to the patient group. Our exclusion criteria for control group were having any health problems, particularly cancer or other chronic diseases, those who do not wish to participate in the study, and living in a different city from the patient group. After obtaining informed consent, the same questionnaire was administered face to face or over the phone to both patient and control groups. In the questionnaire, individuals were asked primarily for demographic information such as age and sex, then smoking habits* (pack/year was asked and grouped as nonsmoker, smokers with a history of 0–5 packs/year, 5–10 packs/year, 10–20 packs/year, and over 20 packs/year)*, and tea consumption* (daily amount of consumption is asked based on the number of cups and one cup was quantified as 80 cc)*.

Traditionally, people start to consume herby cheese from childhood until death. Some of the inhabitants of this region consume herby cheese in three meals a day while some others consume it only in breakfast. And also some people eat only one or two bites at every meal; some of them can eat about 200 to 300 grams. So, to assess the consumption of cheese patients were asked how many meals in which they consumed herby cheese before symptoms have started. And we also asked the control group how many meals they generally consume herby cheese. To assess the amount of cheese consumed in grams, we wanted both groups to think of a matchbox or we showed them a matchbox. Then we asked how much they consumed a meal of this matchbox. We found by weighing that a matchbox of herby cheese is about 50 grams. Finally, we calculated the quantity of herby cheese consumed per day by collecting total amount of cheese consumed per meal.

A tandoor is a cylindrical clay oven used for cooking and baking in many countries like Pakistan, India, Turkey, Iran, Afghanistan, Burma, and Bangladesh. In the east part of Turkey usually dried animal dung is used in tandoor. It is especially used for cooking bread and used only by women. Tandoor exposure was asked based on year* (grouped as no exposure, 0–10 years, 10–20 years, over 20 years exposure)* and recorded.

Pathologic evaluation of the patient group's resection materials was performed after classifying the tumors as esophagus, gastric cardia, corpus, and antrum (esophagus was coded as “0,” cardia “1,” corpus “2,” and antrum “3”) according to localization. Tumor staging was made by using the TNM classification (AJCC 7th edition). Esophageal tumors were divided into two (well differentiated and moderately differentiated squamous cell carcinoma) and gastric tumors into three (well differentiated, moderately differentiated, and poorly differentiated) groups histopathologically. Additionally gastrointestinal stromal tumors and other rarely seen tumors were separately grouped and coded as “GIST” and “Other,” respectively.

Patient group was investigated for the presence of* HP*. When we retrospectively reviewed patients' files, we found that in some cases* HP* status of patients had noted in their endoscopy report and also in their endoscopic biopsy report which had performed before surgery. Another part of patients'* HP* condition was indicated in pathological report after surgery. Unfortunately, we could not find any information about* HP* status in remaining patients. So, we categorized* HP* status as negative (in those* HP* was studied and was negative), positive (in those* HP* was studied and was positive), and unknown (whose* HP* status was not studied or not reported).* HP* diagnosis of all patients, whose* HP* status studied, was made by histological examination after being stained with Giemsa. The data was divided into three groups as unknown, positive, and negative ones.

All data were analyzed statistically with SAS.9.3 programme. For normally distributed data, one-way ANOVA and, for nonnormal distributed data, nonparametric tests Mann-Whitney *U* and Kruskall-Wallis were used for the comparisons between groups. For correlation analysis, Spearman method, for rate comparisons and determining relative risk estimate, chi-square test, and, for calculating cut-off point, logistic regression analysis methods were used. In all analyses, *P* value less than 0.05 was considered statistically significant.

## 3. Results

Patient group comprised a total of 113 esophageal or gastric cancer patients and the control group included 100 subjects. Both groups were similar in terms of sex, number of people, and age (Table 1). Herby cheese (total daily amount in grams) and tea consumptions of the patient and control group were found as indicated in Table 2.

Although there was no significant difference between the groups, hot tea (84°C = temperature in a tea cup) consumption before cooling down, which is one of the habits peculiar to Eastern Anatolia, was found to be too much in both groups (*P* = 0.055).

**Figure 1:**
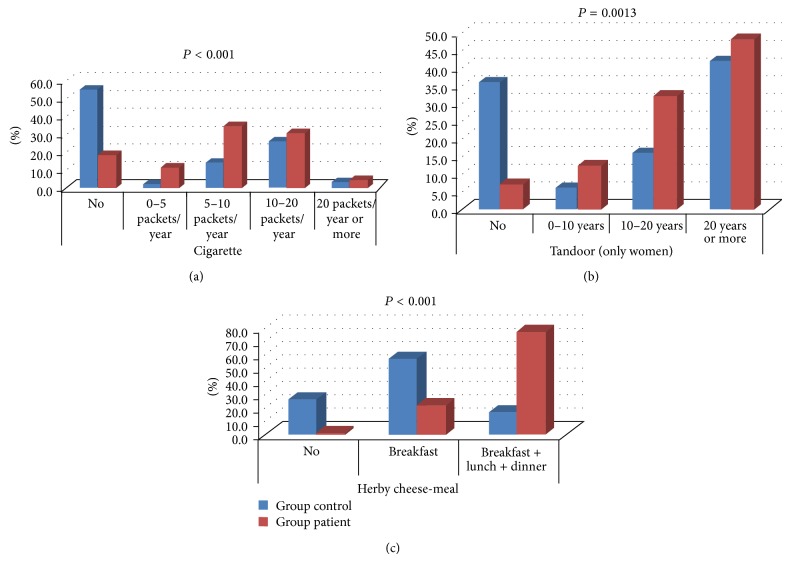
Comparison between the patient and control groups in terms of smoking (a), tandoor exposure (b), and herby cheese consumption per meal (c).

Herby cheese consumption, in both meals and daily total amount in grams, was found to be significantly higher in the patient group (*P* < 0.001). Significant differences were found between the groups in terms of smoking, tandoor exposure, and herby cheese consumption in meals (Figure 1).

When we looked at smoking based on pack-year, it was found that nonsmokers were the majority in control group whereas smokers over 5 packs/year were 65.5% of the patient group. Tandoor exposure was found to be a condition only seen in women in both control and patient groups. Traditionally, cooking bread on tandoor is only made by women. A significant difference was found between female patients and female controls in terms of tandoor exposure (*P* = 0.0013). Nonexposure to tandoor was 7.1% in female patients whereas it was 36.0% in control group. In addition, 48.2% of the women who were exposed to tandoor had more than 20 years of exposure.

**Figure 2:**
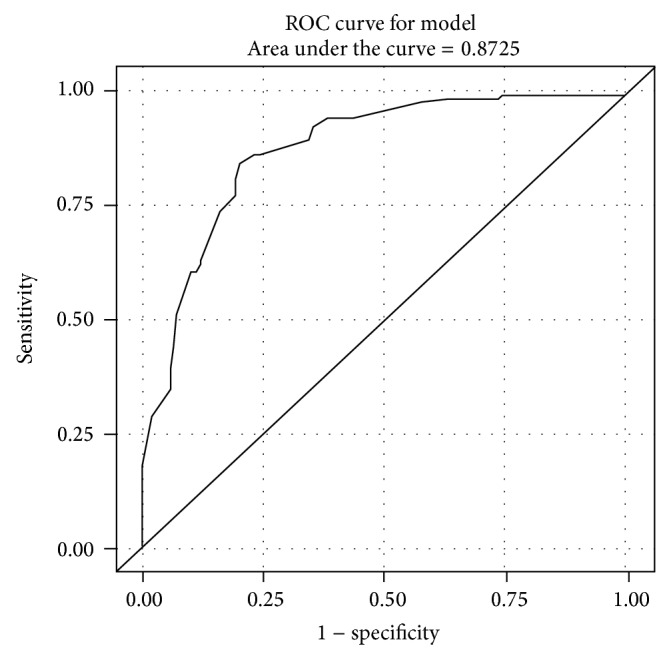
Logistic regression-ROC curve for herby cheese consumption.

**Figure 3:**
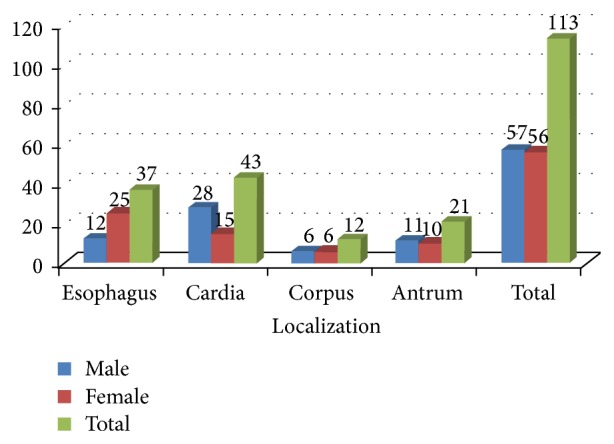
Sex distribution according to tumor localization.

When we considered herby cheese consumption in terms of meals, interestingly we found that individuals in both patient and control groups, who eat herby cheese at breakfast and lunch, always have it for dinner. Therefore individuals consume it either only at breakfast or at 3 meals. 77.0% of the patient group consumes herby cheese at 3 meals whereas this rate was 17% in control group. As a result of the analysis of daily herby cheese consumption (amount in grams) of the groups with logistic regression (Nagelkerke's *R* ^2^ of 0,519) and ROC (Figure 2), it was found that for every 1 gram over 150 gr consumption the risk increases 1.7% (odds ratio: 1.017; 95% Cl: 1.012–1.022). When we looked at the tumor characteristics and eating and lifestyle habits of a total of 113 cancer patients, 37 of the tumors (32.74%) were found to be located in esophagus, 43 (38.05%) in cardia, 12 (10.62%) in corpus, and 21 (18.8%) in antrum. When we looked at the gender distribution according to tumor localization (Figure 3), in 44.64% of the female patients tumors were located in esophagus, 26.79% in cardia, 10.71% in corpus, and 17.86% in antrum whereas these rates were found to be 21.05%, 49.12%, 10.53%, and 19.30%, respectively, in male patients (*P* = 0.0336).

Esophageal cancers were in the majority of female patients whereas in male patients it was found that almost half of the tumors were located in cardia. There was no significant relation between gender and histologic type and stage of the tumor (*P* = 0.86 and *P* = 0.092, resp.). When we compared the tumor localization and stage according to gender differences (Table 3), the distal tumors were found to be diagnosed at a later stage than the proximal tumors.

**Figure 4:**
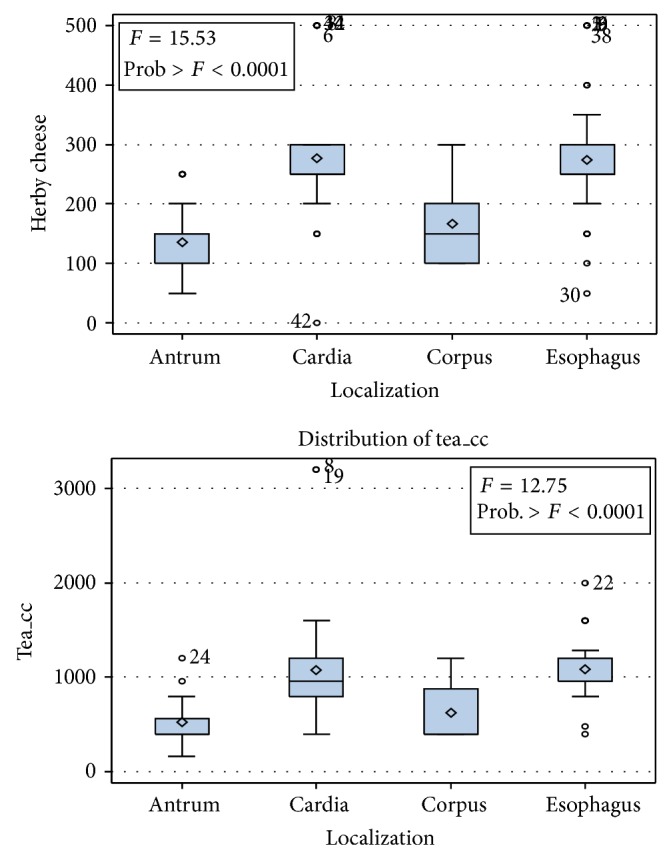
Herby cheese and tea consumption according to tumor localization.

When we looked at the relationship between daily amount of tea and herby cheese consumption and tumor localization (Figure 4), it was found that the amount of consumption was considerably higher in patients with esophagus and cardia tumors than in patients with corpus and antrum tumors (*P* < 0.001). Moderate correlation was detected between the amount of herby cheese and tea consumption and tumor localization (for herby cheese Spearmen rho: −0.50, for tea Spearmen rho: −0.543, *P* < 0.001).

**Figure 5:**
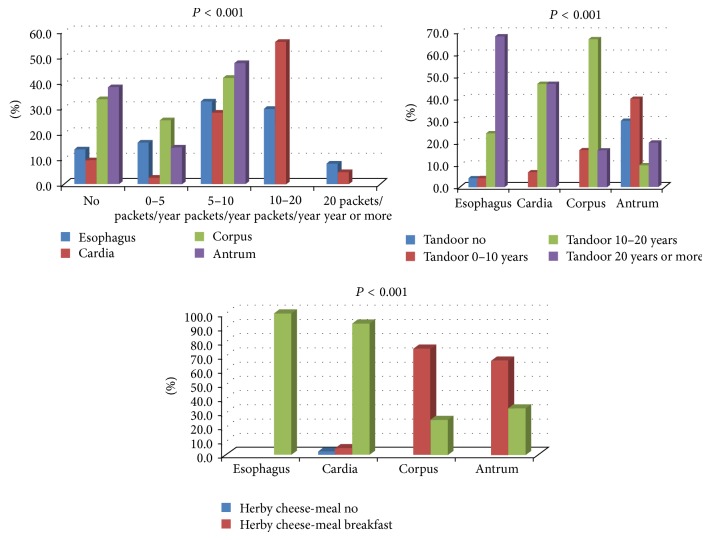
The distribution of smoking, tandoor exposure, and herby cheese consumption per meal according to tumor localization.

It was found that the more distally located tumor the less amount of herby cheese and tea consumption. When we evaluated the tumor localization with the amount of cigarettes smoked, the exposure time to tandoor, and the amount of herby cheese consumption per meal (Figure 5), it was found that all the patients with 10–20 packs/year and more than 20 packs/year history of smoking had esophageal or gastric cardia tumors.

Similarly, the vast majority of the female patients with esophageal or gastric cardia tumors (92% and 93.4%, resp.) were exposed to tandoor for 10–20 years or more than 20 years. All the patients with esophageal tumors were found to consume herby cheese in 3 meals, whereas 93% of the patients with gastric cardia tumors consume it in 3 meals per day. There was a high correlation between herby cheese consumption per meal and tumor localization (Spearman rho: −0.615, *P* < 0.001). Those patients with proximal tumors were found to have more meals.

**Figure 6:**
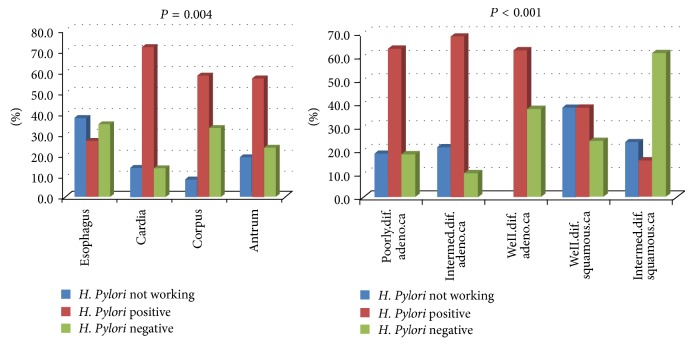
The distribution of* H. pylori* status according to localization of tumor and histopathologic type.

Significant relationship was detected between* HP* positivity and histologic type and localization of tumor (*P* = 0.004 and *P* = 0.01, resp.).* HP* was positive in 72.1% of the gastric cardia tumors. The* HP* positivity ratio was 63.3% in poorly differentiated adenocarcinoma cases and 68.4% in moderately differentiated adenocarcinoma cases whereas it was found to be 15.4% in moderately differentiated squamous cell carcinomas (Figure 6).

## 4. Discussion and Conclusion

According to The World Health Organization (WHO) at least one-third of all cancer cases are preventable and also about 40% of them can be treated with early diagnosis [10]. In some high-risk areas of the world (e.g., Linxina region of China, Golestan province of Iran) the incidence and mortality of esophageal and gastric cancers are about 10 times more common when compared to other low-risk areas [11]. Esophageal and gastric cancers, which rank first among preventable cancers in Van Lake region of eastern Turkey, are still serious public problems just like in those regions mentioned above. In particular common features of some of the eating and lifestyle habits are being emphasized in areas where esophageal and gastric cancers are common [2, 5–9, 12, 13]. Some of these common risks such as low socioeconomic status, heavy cigarette smoking, consuming very hot drinks, high frequency of* HP*, and too salty foods are also common in the Eastern Anatolia whereas indoor cooking bread on tandoor is much more unique for Van Lake region. These risks should be discussed for also Van Lake region to reduce the cancer incidence. In this sense, when we investigated the characteristics of eating and lifestyle habits of Van Lake region, tea consumption at high temperature* (drinking very hot tea before cooling down with a lump of sugar in one's mouth, a drinking style called “kıtlama”)*, heavy cigarette smoking of males from younger ages, consumption of heavily salt-brined herby cheese, and indoor bread cooking on tandoor,* a serious source of carcinogens especially for women*, are all important issues drawing the attention. In this study we investigated the relationship between esophagus and gastric tumors,* which are endemic in Van Lake region*, and those aforementioned eating habits,* which have been the subject of many studies*; there were no statistically significant effects of those hot drinks emphasized in the literature. However, it is an undeniable fact that drinking at a temperature of approximately 84°C with an amount of up to1 liter of black tea per day can cause thermal injury to the esophagus. Islami et al. reported in their systemic study that there is little evidence showing the relationship between the amount of tea and coffee consumption and esophageal cancer whereas there are strong evidences showing that the temperature of the drinks increases the cancer risk [7]. We considered that the habit of drinking very hot tea right after pouring the cup, with a lump of sugar in one's mouth,* so-called “kıtlama type drinking” in the region*, exposes the esophagus to high temperatures.

It is widely accepted in both western and eastern studies that smoking increases the risk of esophageal and gastric cancer [14, 15]. We believed that heavy cigarette smoking is much more common among men whereas, for women, dense smoke, released from dung cake (called “tezek” in the region) burning when used for tandoor fuel, is a considerable risk. Squamous cell carcinoma is 2-3 times more common in males than females in low-risk areas of the world whereas these ratios become equal in some of the regions of China and Iran,* known as high-risk areas* [14, 16]. However, interestingly, in our study, esophageal tumors were found to be 2 times more common in females than males in contrast to the literature. In this case, we thought that tandoor exposure, which is seen only in females, might have role. Furthermore, in our study, it was reported that approximately 66% of the female patients with esophageal cancer were exposed to tandoor for more than 20 years.

Traditionally, herby cheese is heavily salt-brined and it is consumed after waiting for 3-4 months in the region. High amounts of salt, nitrate and nitrite, which are found in brined foods and also in herby cheese, are known to be possible risks for esophageal and gastric cancers [17]. Previously, in the study of Türkdoğan et al. in Van Lake region, the detected amount of nitrate and nitrite was found to be significantly higher in herby cheese in comparison with normal white cheese [9]. In our study we reported that more than 150 gr herby cheese consumption per day increases the cancer risk (*P* < 0.001) and majority of the patient group (>70%) consumes it 3 meals per day. These findings, when considered together with the exposure to high levels of nitrous components, might be important in cancer etiopathogenesis.

Since the* HP* was accepted as a biological carcinogen by WHO, it has been reported in many studies in the literature that* HP* positivity increases the risk of gastric (especially the distal tumors) cancer [18, 19]. When socioeconomic status of the Van Lake region and unawareness and poor hygiene status of the inhabitants are taken into consideration together with the fact that very low levels of* HP *eradication are applied in the region, how risky the* HP* is for the region can be estimated. In this study* HP *was present in 68% of the cancer cases. In particular in cardia located cancers positivity rates were too high far beyond the literature [19]. Although there are studies indicating that* HP* positivity decreases the risk of esophageal cancer, debates in this issue still continues [14, 20]. In parallel with this information, also in our study, we found that* HP* positivity was significantly lower compared to other localizations (*P* = 0.004).

As with all retrospective case-control studies, the most important limitations of our study were the subjects' mistakes in remembrance of the data and prejudices of the data collectors. Nevertheless, we believe that noticing the negative relationship between lifestyle and eating habits and esophageal and gastric cancers in Van Lake region and taking community-based measures towards these problems can decrease the incidence and mortality rates of these tumors although not reaching the rates of WHO.

## Figures and Tables

**Table 1:** Age and sex distribution of the patient and control groups.

	Patient group		Control group		*P*
	Male	Female	Male	Female	
*N*	57	56	50	50	
Age					
(Mean ± Std. dev.)^*^	59.1 ± 10.8	59.64 ± 11.1	57.82 ± 12.3	60.9 ± 11	0.984
(Min–Max)^**^	(32–83)	(34–83)	(27–80)	(38–83)	

^*^Mean age and standard deviation.

^**^Minimum and maximum ages.

**Table 2:** Comparison between patient and control groups in terms of herby cheese and tea consumption.

	Patient group	Control group	*P*
	Mean (%95-CI)^*^	Mean (%95-CI)^*^	
Amount of Herby cheese (gram/day)	238.05 (217.85–258.26)	85.00 (68.71–101.29)	<0.001
Tea (cc/day)	929.38 (841.49–1017.28)	1045.70 (966.04–1125.36)	0.055

^*^Mean and 95% confidence interval.

**Table 3:** The distribution of tumor stage and localization according to sex in patient group.

		Stage									Total	
		Stage 0	Stage 1a	Stage 1b	Stage 2a	Stage 2b	Stage 3a	Stage 3b	Stage 3c	Stage 4		
Male	Esophagus	0 (%0)	0 (%0)	1 (%8.3)	3 (%25)	3 (%25)	1 (%8.3)	1 (%8.3)	0 (%0)	3 (%25)	12 (%100)	*P* = 0.298^*^
	Cardia	0 (%0)	1 (%3.6)	0 (%0)	1 (%3.6)	9 (%32.1)	4 (%14.3)	5 (%17.9)	3 (%10.7)	5 (%17.9)	28 (%100)	
	Corpus	0 (%0)	1 (%16.7)	0 (%0)	0 (%0)	2 (%33.3)	0 (%0)	0 (%0)	0 (%0)	3 (%50)	6 (%100)	
	Antrum	0 (%0)	0 (%0)	1 (%9.1)	0 (%0)	4 (%36.4)	2 (%18.2)	2 (%18.2)	0 (%0)	2 (%18.2)	11 (%100)	

Female	Esophagus	1 (%4)	1 (%4)	5 (%20)	9 (%36)	4 (%16)	1 (%4)	2 (%8)	0 (%0)	2 (%8)	25 (%100)	*P* = 0.127^*^
	Cardia	0 (%0)	0 (%0)	0 (%0)	1 (%6.7)	6 (%40)	2 (%13.3)	3 (%20)	1 (%6.7)	2 (%13.3)	15 (%100)	
	Corpus	0 (%0)	0 (%0)	1 (%16.7)	1 (%16.7)	1 (%16.7)	0 (%0)	2 (%33.3)	0 (%0)	1 (%16.7)	6 (%100)	
	Antrum	0 (%0)	0 (%0)	2 (%20)	1 (%10)	0 (%0)	2 (%20)	1 (%10)	2 (%20)	2 (%20)	10 (%100)	

^*^No relationship was found between tumor stage and localization of males and females when interpreted separately.
